# Integrated analysis of usnic acid as a potential inhibitor of dengue NS2B/NS3 protease: DFT, molecular docking, MEP, ADMET, and drug-likeness evaluation

**DOI:** 10.5114/bta/216303

**Published:** 2026-05-22

**Authors:** Neni Frimayanti, Ihsan Ikhtiarudin, Maydhea Syakirah

**Affiliations:** Sekolah Tinggi Ilmu Farmasi Riau, Pekanbaru, Riau, Indonesia

**Keywords:** density functional theory, dengue DENV-2 NS2B/NS3, molecular docking, usnic acid

## Abstract

**Background:**

The present study aimed to evaluate the inhibitory capability of 10 pyrazole-usnic acid derivatives against dengue virus serotype 2 (DENV-2) NS2B/NS3 serine protease. The crystallographic structure model of the protease enzyme (PDB code: 2FOM) was used to conduct molecular docking studies of pyrazole-usnic acid derivatives with target proteins. Computational analysis was performed using the Molecular Operating Environment program, Gaussian View software, and ADMETLab platform.

**Materials and methods:**

The inhibitory potential of the derivatives was tested by molecular docking using panduratin A as the positive control, together with density functional theory, molecular electrostatic potential analysis, drug-likeness assessment, and ADMET (absorption, distribution, metabolism, excretion, and toxicity) profiling.

**Results:**

Among the tested derivatives, compounds 5 and 6 exhibited the most promising inhibitory effects against DENV-2 NS2B/NS3 serine protease. The binding free energy values for these compounds were –7.320 and –7.477 kcal/mol, respectively. These two compounds shared multiple amino acid residues with panduratin A, which was used as a reference inhibitor. Compounds 5 and 6 also displayed negative electrostatic regions surrounding their oxygen atoms, with gap energies of 0.133 and 0.137 eV and dipole moment values of 3.237 and 2.806 D, respectively.

**Conclusions:**

These computational findings suggest that compounds 5 and 6 may serve as preliminary candidates for dengue virus inhibition. However, further clinical and experimental studies are required to confirm their efficacy and safety.

## Introduction

*Aedes aegypti* is the primary vector of dengue transmission. The illness can manifest as mild flu-like symptoms or progress to more severe conditions such as dengue hemorrhagic fever and dengue shock syndrome. Typical symptoms of dengue include elevated body temperature, severe headache, ocular discomfort, musculoskeletal pain, skin eruptions, and minor bleeding. In extreme cases, delayed medical intervention may result in substantial hemorrhage, circulatory collapse, and vascular leakage, possibly leading to patient’s death. Dengue is common in tropical and subtropical areas worldwide, particularly in Southeast Asia, the Pacific Islands, the Caribbean, and certain regions of Central and South America. Mosquito breeding increases during rainy periods, leading to higher rates of dengue transmission. Currently, there is no specific antiviral therapy for dengue, and treatment primarily focuses on symptom management and fluid replacement. Preventive measures emphasize individual protection from mosquito bites and mosquito population control to reduce the risk of infection. Although dengue vaccines are accessible in some areas, their administration is governed by local immunization policies and epidemiological guidelines (Lessa et al. 2023; Kularatne et al. 2022).

Usnic acid, a secondary metabolite produced by various lichen species, has attracted increasing interest because of its wide-ranging pharmacological properties, including antiviral effect (Filimonov et al. 2022), antimicrobial effect (Francolini et al. 2004), and anti-inflammatory effect (PaŸdziora et al. 2023). The therapeutic potential of natural compounds has been enhanced by recent advances in computational techniques. Molecular docking studies and density functional theory (DFT) have been instrumental in elucidating the binding interactions between potential inhibitors and their target proteins (Celik 2023; Abu-Izneid et al. 2024).

The present study aimed to assess the safety and effectiveness of usnic acid as an inhibitor of dengue virus serotype 2 (DENV-2) NS2B/NS3 protease and to advance the development of novel dengue fever treatment by using computational methodologies. For this purpose, we investigated the binding affinity of usnic acid to DENV-2 NS2B/NS3 protease using a multifaceted approach involving DFT, molecular docking, and molecular electrostatic potential (MEP) analyses. To assess the potential of usnic acid as a therapeutic candidate, we evaluated its drug-likeness properties and absorption, distribution, metabolism, excretion, and toxicity (ADMET) profile.

## Materials and methods

### Molecular docking

Panduratin A, a naturally occurring chalcone derivative from *Boesenbergia rotunda*, was used as the reference compound, and 10 ligands were created using ChemDraw 15.0. Panduratin A can inhibit DENV-2 NS2B/NS3 protease and therefore was selected as the positive control. It binds to critical protease catalytic residues (such as His51, Asp75, and Ser135) with favorable docking scores and binding modes. It also shows antiviral activity. Panduratin A is considered a trustworthy benchmark for comparative analysis in dengue antiviral research because of its well-defined interaction profile and frequent application in previous computational and experimental investigations.

These ligand structures were first imported into Discovery Studio Visualizer before their transfer to the Molecular Operating Environment (MOE) 2022.0901 software package developed by the Chemical Computing Group. To facilitate their use in docking simulations, ligand files were integrated into the mdb database within the MOE software. The ligands and reference compounds are listed in Table 1.

**Table 1:**
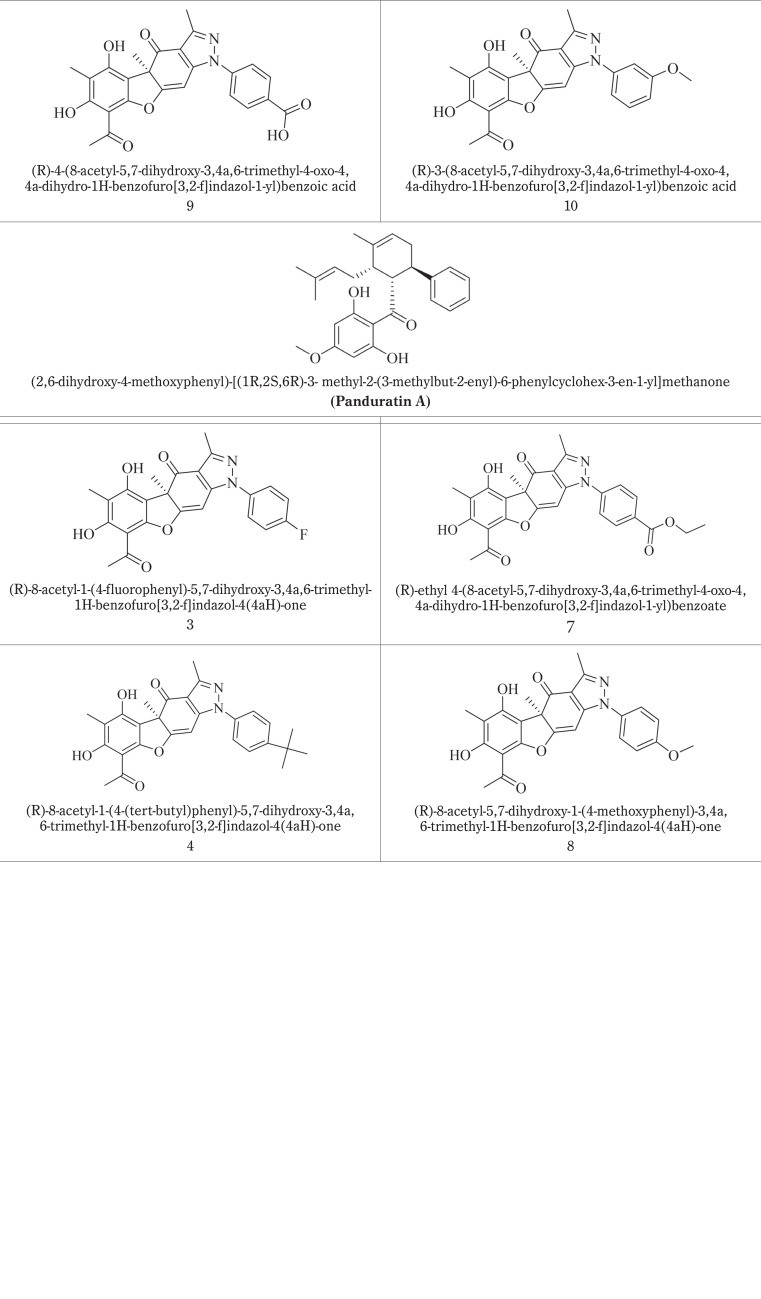
Molecular structure of ligands

The protease protein (PDB ID: 2FOM) was acquired from the RCSB Protein Data Bank. To prepare the protein for molecular docking, hydrogen atoms were added, and the initial ligand was removed from the protein structure. Structural validation of the protein model was conducted, including the identification of missing loops and the assessment of key structural motifs. Loop modeling and refinement were performed using Structural Analysis and Verification Server SAVES v6.1 (https://saves.mbi.ucla.edu/). The refined structure was subjected to energy minimization using the CHARMM27 force field, focusing on the alpha carbon and backbone atoms. An RMS gradient of 0.01 was utilized to reduce the energy of the backbone, alpha carbon, and hydrogen atoms.

Following minimization, the protein structure was saved in the PDB format. Before docking, the potential binding sites were identified using a site finder tool to detect active regions on the protein surface. The third site was selected for docking studies. To confirm this selection, structural analysis was performed using CASTp (http://sts.bioe.uic.edu/castp/index.html?1bxw). CASTp validated that the selected third site included amino acid residues suitable for ligand binding. Furthermore, protonational state prediction for the protein was performed at pH 7, and the electrostatic interaction was also applied using the Generalized Born Volume Integral model.

During the docking setup, the selected binding site was marked with a dummy atom, and ligand configurations were imported from the MDB file. The docking process was performed using rigid refinement, with posture configurations of 100 and 30 and a triangular placement method. Docking simulations were initiated following the completion of these steps.

### Molecular dynamics

Molecular dynamics (MD) simulations were performed using complexes formed between the ligand (compounds 5 and 6) and serine protease. The protein, with a resolution of 1.5 Å, was obtained from the Protein Data Bank (PDB ID: 2FOM). Nanoscale Molecular Dynamics software version 2.9 was used to perform these preliminary investigations. The CHARMM27 (Chemistry at Harvard Macromolecular Mechanics) force field was selected because of its appropriateness, and this force field was applied for MD simulations of each compound. A TIP3P water box featuring a 2.5 Å water layer in all dimensions was implemented to replicate the protein environment (Nurulita et al. 2021; Frimayanti et al. 2021).

The system underwent a gradual thermal increase from 0 to 300 K over 100 ps using an canonical ensemble (NVT, constant number of particles, volume, and temperature). Following this initial phase, MD simulations were conducted within an isothermal–isobaric ensemble (NPT, constant number of particles, pressure, and temperature), incorporating periodic boundary conditions for 150 ns, while ensuring that temperature and pressure coupling occurred at 1-ps intervals. Spatial coordinates were sampled every 0.1 ps to facilitate binding free energy calculations and conformational analysis. Following completion of the heating and equilibration processes, a 150-ns production MD run was executed within the NPT ensemble.

### DFT

By using DFT, we conducted computational simulations utilizing the B3LYP hybrid functional, 6-31G basis set, and Lee-Yang-Parr correlation functional through Gauss View 5 software. This study aimed to optimize the molecular structures, perform frequency analyses, and create MEP maps for each molecule. Prior to optimization, we explored potential molecular configurations using conformational analysis. Frequency calculations were performed to ensure that the optimized structures represented true minima.

### MEP

MEP maps were generated to assess the electrostatic surface properties of the studied molecules. These visual representations depict the electrostatic potential distribution surrounding a molecule, which is essential for understanding its reactivity and possible interactions with other compounds. First, the molecular structures were optimized using the DFT/B3LYP method in conjunction with the 6-31G (d,p) basis set by utilizing Gauss View 5 software. Subsequently, MEP calculations were performed using the same program as mentioned above. The resulting MEP maps were displayed using Gauss View 5.0, facilitating a comprehensive analysis of the electrostatic potential on the surfaces of the molecules. By examining these maps, we identified regions of positive and negative electrostatic potentials, indicating potential sites for intermolecular interactions. This analysis contributes to the understanding of the behavior of the molecules in various environments and their interactions with other chemical entities.

### ADMET profiling and drug likeness

To assess the physicochemical and pharmacokinetic properties, including ADMET parameters, the following two web-based platforms were utilized: admetSAR 2.0 (http://lmmd.ecust.edu.cn/admetsar2/admetopt/, accessed March 23, 2024) (Jia et al. 2020) and ADMETlab 2.0 (https://admetmesh.scbdd.com/service/evaluation/index, accessed April 20, 2024) (Xiong et al. 2021). Drug-likeness and oral bioavailability were evaluated using Lipinski’s rule, which suggests potential suitability when compounds comply with the “rule of 5.” This rule stipulates that molecules should have a molecular weight (MW) of less than 500 g/mol, no more than 10 hydrogen bond acceptors (nHA), a maximum of five hydrogen bond donors (nHD), and a logarithm of the n-octanol/water partition coefficient (logP) of ≤ 5 (Lipinski et al. 2001).

## Results

### Molecular docking

**Figure 1:**
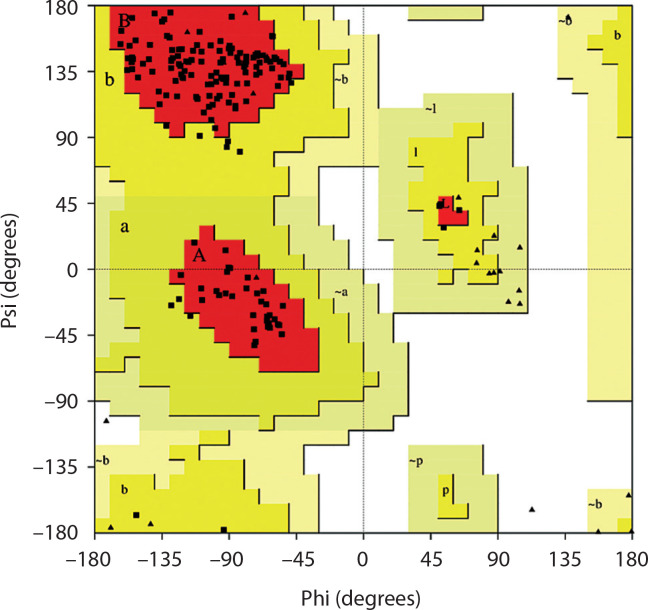
Ramachandran plot for the protein 2FOM

The Ramachandran plot is a crucial tool in protein structure analysis, enabling validation of protein conformations and offering insights into their structural features. The Ramachandran plot for the NS2B-NS3 protease enzyme shows that the majority of amino acid residues are located within the favored regions (Figure 1), which signifies structural stability; conversely, a limited number of outliers imply a degree of flexibility.

Table 2 shows the docking outcomes for these ligands, with panduratin A as the positive control. Molecular docking, a popular computational method in drug discovery, serves two primary functions: predicting the behavior of new chemical compounds and identifying potential inhibitors by examining their interactions with the target protein. This approach offers crucial information on ligand binding strength and selectivity, facilitating the creation and improvement of novel therapeutic agents. In this study, the ideal docking configuration was selected based on the following two specific criteria: minimum binding free energy and root mean square deviation (RMSD) below 2 (Frimayanti et al. 2023; Frimayanti et al. 2024b).

**Table 2:** Docking results

Compound	Binding free energy [kcal/mol]	RMSD	H-bond	Hydrophobic	van der Waals	Other interaction	Binding factor
Positive control (Panduratin A)	–5.481	1.144	His51 Pro132	–	Asp75	Leu128, Ser135, Phe130, Ser131, Tyr161, Gly151, Gly153, Asn152	11
1	–6.173	1.113	His51, Gly153	Arg54	Asp75	Val72, Lys73, Asn152, Leu126 Gly151 Ser135	6
2	–7.030	1.104	–	–	Asp75	Asn152, Val154, Gly153, Gly151, Leu128, His51, Tyr150, Phe130, Ser135, Ser131, Pro132, Tyr161, Met49	11
3	–7.038	1.191	Gly153	–	Asp129	His51, Ser135, Gly151, Tyr150, Phe130, Leu128, Pro132, Ser131, Tyr161	9
4	–6.851	1.767	Phe130	–	Asp75, Asp129	Gly153, Val154, Asn152, His51, Ser135, Pro132, Ser131, Tyr150, Phe130, Leu128	10
5	–7.320	0.922	Gly153, His51, Asp129	–	Asp75, Asp129	Ser135, Gly151, Ser131, Tyr150, Pro132, Phe130, Leu128, Tyr161	10
6	–7.477	1.858	His51, Gly153, Leu128	–	Asp75, Asp129	Gly151, Asn152, Ser135, Phe130, Pro132, Tyr161, Ser131	11
7	–7.166	1.776	Leu128	–	Asp75	Asn152, His51, Tyr150, Gly151, Gly153, Ser135, Phe130, Pro132, Ser131, Tyr161	11
8	–7.490	1.480	His51	–	Asp129	Val154, Asn152, Gly153, Gly151, Ser135, Pro132, Leu128, Phe130, Tyr161	9
9	–7.470	0.844	Gly153, His51	–	Asp129	Ser135, Ser131, Pro132, Phe130, Tyr161, Tyr150, Gly151, Leu128, Asn152	10
10	–7.519	1.821	Leu128, Pro132, Phe130	–	Asp75, Asp129	His51, Met49, Asn152, Val154, Gly153, Gly151, Ser135, Tyr150, Ser131, Tyr161	11

RMSD – root mean square deviation.

### MD simulations

MD simulations were conducted on the protease protein (2FOM) together with panduratin A as the positive control. The analysis revealed that this protein exhibited interactions with residues His51 and Pro132 through hydrogen bonding interactions before and after MD simulations. MD simulations were conducted on the protein-ligand complex comprising panduratin A, compound 5, and compound 6. The results indicated that compounds 5 and 6 engaged with the amino acid residues Gly153, His51, Asp129 and Leu128, respectively, through hydrogen bonding. The interaction between compounds 5 and 6 was also elucidated with reference to the hydrogen bond distance, which was less than 2.9 Å. The interactions between the positive control and selected compounds before and after MD simulations are presented in Table 3.

**Table 3:** The molecular binding and molecular interaction during molecular dynamics (MD) simulation

Compound	After docking	MD simulation	Distance of hydrogen bond interaction	Angles of hydrogen bond interaction
Compound 5	Gly153, His51, Asp129	Gly153, His51, Asp129	2.9 Å	1570
Compound 6	His51, Gly153, Leu128	His51, Gly153, Leu128	2.9 Å	1600
Panduratin A	His51, Pro132	His51, Pro132	2.9 Å	1520

**Figure 2:**
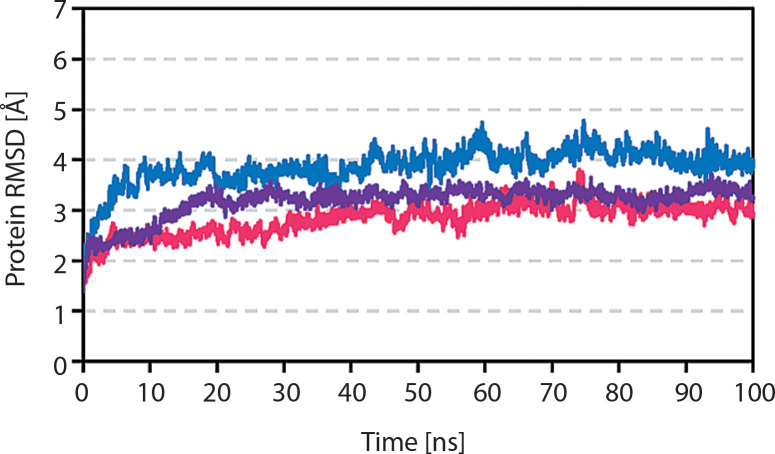
Root mean square deviation (RMSD) values for panduratin A-protein complex (purple), compound 5-protein complex (blue), and compound 6-protein complex (pink)

The RMSD value was used to evaluate the dynamic characteristics of the complex. The conformational stability of the complex, which serves as a structural and dynamic parameter, was assessed using the RMSD value. An elevated RMSD value indicates reduced protein stability. Based on this analysis, the compound 5-protein complex exhibited oscillatory behavior at 70 ns, with an average RMSD of 4.5 Å. Prior to the 40 ns mark, the compound 5-protein complex exhibited some fluctuations in the mean RMSD value; however, it remained consistent throughout the remainder of the simulation. This complex also demonstrated an average RMSD of **~**4.5 Å. In contrast, the compound 6-protein complex oscillated for 75 ns with an average RMSD of 0.35 Å. This complex exhibited stability and robust bonding, as evidenced by its low average RMSD value and minimal volatility, which were nearly indistinguishable. A reduced RMSD value was associated with enhanced compound stability. Figure 2 shows the RMSD value of compound 5.

### DFT

To refine the molecular configurations and explore energetically favorable conformations without considering solvent interactions, gas-phase structure optimizations were conducted using DFT calculations for compounds 5 and 6. These compounds were selected based on their superior performance in molecular docking studies, which indicated that they possessed the lowest binding free energy among the examined candidates.

### MEP

**Figure 3:**
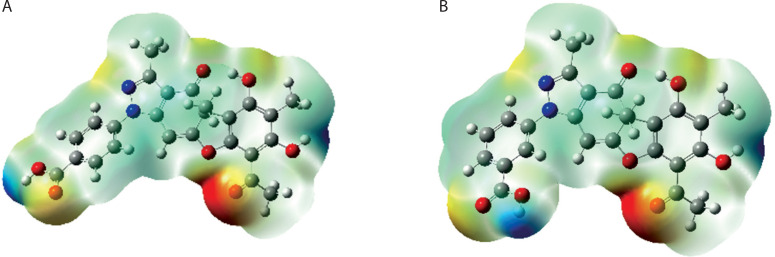
Molecular electrostatic potential for (**A**) compound 5 and (**B**) compound 6. Color gradient denotes electrostatic potential (electron volts – eV), with red indicating regions of high electron density and blue indicating regions of low electron density. This figure highlights potential electrophilic and nucleophilic reaction sites with a visible color scale for reactivity interpretation

MEP is a crucial indicator to evaluate the reactivity of a molecule toward nucleophilic and electrophilic agents. It offers insights into the distribution of nuclear and electronic charges within the molecule. The MEP analysis uses a color-coded visual representation to illustrate the electrostatic potential throughout a molecule, with blue denoting positive potential, red indicating negative potential, and green representing neutral potential (Kavita et al. 2024; Mary et al. 2021). Regions colored blue or red signify the preferred locations for nucleophilic or electrophilic attack, respectively, as positively charged areas attract nucleophilic species, whereas negatively charged areas attract electrophilic species. MEP surfaces for compounds 5 and 6, which exhibited potent dengue inhibitory activity, were generated using DFT-optimized geometries and are displayed in Figure 3.

### ADMET profiling and drug likeness

The drug-likeness concept evaluates the physicochemical properties of a compound in relation to its expected biopharmaceutical effect in the human body. According to Benet et al. (2016) and Erol et al. (2021), Lipinski’s rule of five provides a framework to assess whether a bioactive compound could be considered a drug candidate. This rule includes four primary criteria: (1) MW < 500, (2) octanol-water partition coefficient (log P) ≤ 5, (3) maximum number of nHD = 5, and (4) maximum number of nHA = 10. ADMET encompasses crucial aspects of pharmaceutical profiling to determine a drug’s appropriateness for human consumption. Table 4 presents the drug-likeness and ADMET characteristics of compounds 5 and 6.

**Table 4:** Density functional theory results

Compounds	Energy	Electronic structure		Energy gap
		HOMO	LUMO	
5	–1600.313	–0.225	–0.092	0.133
6	–1600.312	–0.223	–0.086	0.137

HOMO – highest occupied molecular orbital, LUMO – lowest unoccupied molecular orbital.

## Discussion

This study evaluated the potential of usnic acid compounds as dengue inhibitors by using computational methods to analyze their binding affinities. We used MOE 2022.0901 software to examine the interactions between the ligands and the protease enzyme. The docking results were benchmarked against panduratin A, which was used as a positive control. This methodology facilitated the understanding of how the compounds interact with the active site of the target protein.

Before conducting molecular docking investigations, the precision and integrity of the protein model were meticulously assessed through SAVES v6.1 (accessible at https://saves.mbi.ucla.edu/). This comprehensive platform integrates the appraisal of the stereochemical fidelity and dependability of the modeled protein conformation. Crucial validation parameters, including the distribution of the Ramachandran plot, overall model quality factor, and compatibility between the 3D and 1D profiles, were reviewed to ascertain that the protein model conformed to the requisite standards for precise docking procedures. These findings substantiate the structural authenticity of the model, thereby validating its application in subsequent molecular docking simulations and ensuring the credibility of the interaction predictions.

**Figure 4:**
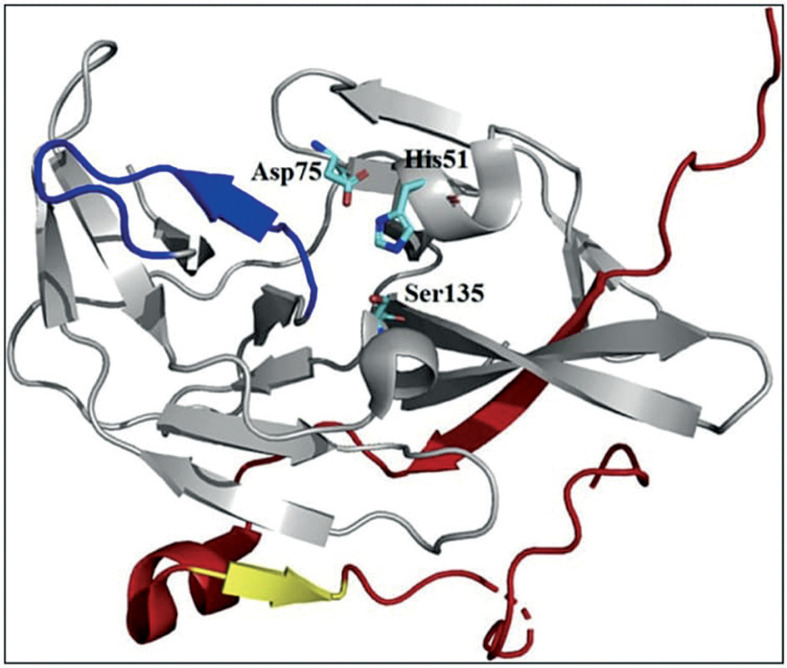
X-ray crystallographic analysis of the catalytically favorable conformation of dengue virus serotype 2 (DENV-2) NS2B/NS3 protease (Protein Data Bank ID: 2FOM). The grey ribbon delineates the structure of NS3, the red ribbon represents the NS2B cofactor, the yellow ribbon signifies the S1-β-hairpin, and the blue ribbon illustrates the ST-loop (Erbel et al. 2006)

NS3 is a substantial multifunctional protein characterized by serine protease activity (with NS2B acting as a cofactor) and capabilities identical to those of 5′-RNA triphosphatase, nucleoside triphosphatase, and helicase (Wengler 1991; Warrener et al. 1993; Li et al. 1999). The N-terminal segment of NS3, comprising 170 amino acids, exhibits protease functionality and carries NS2B – a hydrophobic core of approximately 40 amino acids, which is integral to its role as a cofactor (Chambers et al. 1990; Chambers et al. 1991; Falgout et al. 1991).^.^ NS3 protease (NS3 pro) is a trypsinlike serine protease characterized by a classical serine protease catalytic triad comprising His51, Asp75, and Ser135 residues (Bazan and Fletterick 1989). All four DENV serotypes exhibit an approximate amino acid sequence homology of 65–74%, along with a shared substrate preference (Li et al. 2005). The C-terminal β-hairpin of NS2B envelops the active site of NS3 in its catalytically active conformation (Figure 4) (Erbel et al. 2006). In line with the critical structural function of the C-terminal β-hairpin of NS2B, comparative structural analyses revealed that the amino acids in the N-terminal region exhibited analogous conformations across all structures, regardless of the presence of inhibitors.

Table 2 shows the energy values of molecular docking studies. The findings revealed that all compounds demonstrated energy values lower than those of the positive control panduratin A. Among all compounds, compounds 5 and 6 showed the most favorable binding free energy (–7.320 and –7.477 kcal/mol, respectively). In contrast, panduratin A had a binding free energy of –5.481 kcal/mol. Notably, lower binding energy values typically indicate a greater probability of ligand-receptor interactions.

The molecular docking data revealed that compounds 5 and 6 demonstrated strong anticipated interactions with the DENV-2 NS2B/NS3 protease, as indicated by their reduced binding free energy values (–7.320 and –7.477 kcal/mol, respectively) compared to that of the positive control panduratin A (–5.481 kcal/mol). These findings imply that compounds 5 and 6 have a greater propensity to bind to the active site of the target protein. Notably, both compounds interacted with vital catalytic residues necessary for protease activity, including His51 and Asp75, respectively. Their potential as inhibitors is further supported by the strong binding factor, which shows an overlap with the positive control in terms of binding site residues. Additionally, the possibility of sustained and specific binding is increased by hydrogen bonding interactions, particularly with residues such as Gly153, Ser135, and Phe130. These docking parameters support the hypothesis that compounds 5 and 6 could be promising initial candidates for dengue virus suppression by efficiently binding to the NS2B/NS3 protease active site (Ipek et al. 2023; Frimayanti et al. 2024a).

It should be noted that molecular docking offers only theoretical predictions of the manner and strength of ligand-protein binding. Although these findings do not validate the biological activity of the molecules, they indicate potential molecular links. Therefore, to confirm the antiviral effectiveness and safety of these drugs, experimental validation through *in vitro* and *in vivo* studies is crucial. According to docking simulations, compound 5 forms hydrogen bonds with Gly153 (through its phenyl group), His51 (through its CH group), and Asp129 (through its hydroxyl group) to interact with the DENV-2 NS2B/NS3 protease. A possible inhibitory effect was suggested by interactions with the catalytic residues His51 and Asp75, which are crucial for protease activity. Furthermore, the stability of the ligand-protein complex may be supported by van der Waals interactions with residues Asp75 and Asp129. It is critical to note that these results are computational predictions and that the biological activity of the compounds requires validation through experimental research.

**Figure 5:**
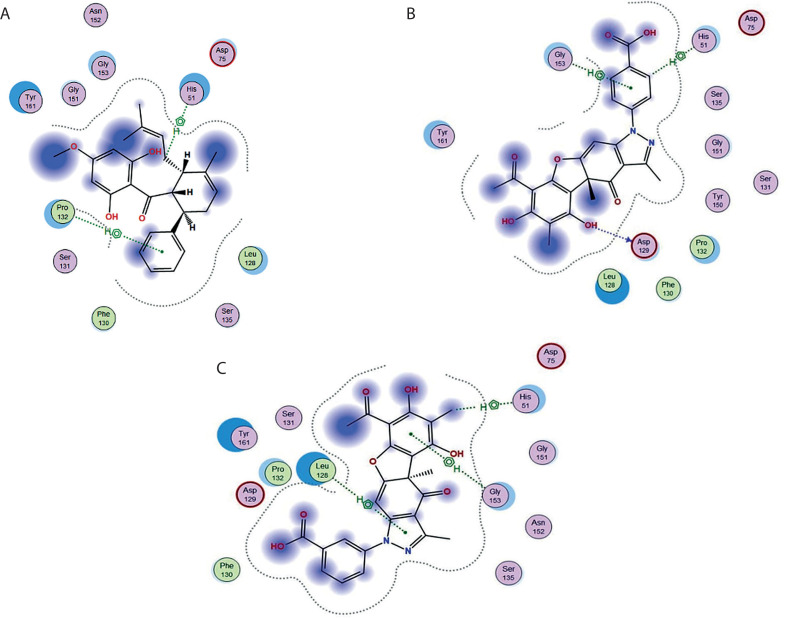
Spatial arrangement of (**A**) panduratin A, (**B**) compound 5, and (**C**) compound 6

Compound 6 exhibited hydrogen bonding with several amino acid residues: His51 on the carbon group, Gly153 on the phenyl group, and Leu128 on the carbonyl group. This compound also formed van der Waals interactions with the amino acid residues Asp75 and Asp129. These van der Waals forces significantly enhance the ability of these compounds to bind to proteinbinding sites. These observations highlight the complex nature of ligand-protein interactions and suggest that these compounds can function as potent inhibitors through various binding modes. The inhibitory effects of these compounds may be attributed to such interactions. Figure 5 illustrates the three-dimensional arrangements of these compounds.

Based on MD simulations, these compounds could sustain the presence of hydrogen bonds, with a hydrogen bonding distance of 2.9 Å (Bursch et al. 2022). The presence of hydrogen bonding in both compounds 5 and 6 suggests that these compounds are bioactive.

The gas-phase molecular structures of compounds 5 and 6 were optimized by DFT and B3LYP methods, utilizing the 6-31G basis set in Gaussian View software 5.0 (Ipek et al. 2023). To evaluate the chemical stability of these compounds, the highest occupied molecular orbital (HOMO) and lowest unoccupied molecular orbital (LUMO) were examined. The DFT/B3LYP/6-31G approach was used to determine the energy gap and reactivity descriptors (Ipek et al. 2023).^.^ Table 4 presents the results of these analyses.

**Figure 6:**
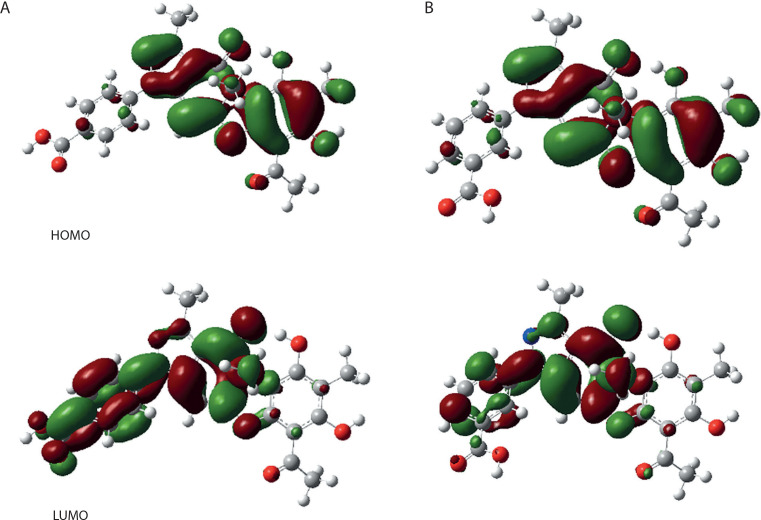
The highest occupied molecular orbital (HOMO) and lowest unoccupied molecular orbital (LUMO) values for (**A)** compound 5 and (**B**) compound 6. Diagrams display molecular orbitals with color coding for phase difference. Energy levels are annotated in electron volts (eV) to represent the HOMO-LUMO energy gap, indicating reactivity potential

The research findings indicate that compounds 5 and 6 have energy gaps of 0.133 and 0.137 eV, respectively. The minimal difference between the LUMO and HOMO values suggests that these compounds are chemically unstable and have low stability, making them more prone to be affected by environmental disturbances. This property contributes to their high biological activity. The electron-donating capacity of a compound is represented by the energy of the HOMO, while its electron-accepting ability is denoted by the energy of the LUMO. Figure 6 illustrates the HOMO, LUMO, and energy gaps of compounds 5 and 6. These findings were corroborated by the results of molecular docking studies.

The maximum red intensity shown by a molecule indicates the presence of a negative electrostatic potential, which is dispersed across all oxygen atoms. This distribution greatly increases the vulnerability of the hydrogen atoms to nucleophilic attacks, while the oxygen atoms become prime targets for electrophilic attacks. The electrostatic potential distribution differs among molecules or drugs because of the specific types and electronic properties of their constituent atoms. Consequently, the varying patterns of the electrostatic potential of a drug may offer insights into its biological activity (Burch et al. 2022).

MEP analysis of compounds 5 and 6 revealed distinct electrostatic regions. The oxygen atoms, particularly those in the ketone group, exhibited a negative electrostatic potential, which was visualized as red areas. Conversely, positive electrostatic regions (blue) were observed around the hydrogen atoms, particularly those bonded to hydroxyl groups. These electronic characteristics influence the interaction between the drug and its receptor. These findings suggest that the oxygen atom in both compounds 5 and 6 serves as the most suitable site for drug-receptor binding.

We used Lipinski’s rule to assess the physicochemical characteristics of all compounds, including MW, nHA, nHD, and n-octanol/water partition coefficient logarithm (logP). Based on these criteria, compounds 5 and 6 are anticipated to be orally viable and to comply with Lipinski’s rule. These two compounds exhibited lower logP values than panduratin A, suggesting enhanced hydrophilicity. The human intestinal absorption score indicated favorable drug absorption, with a predicted value of 0.01 (ranging from 0 to 0.3) (Suzuki et al. 2011).

Drug permeability was evaluated using human colon adenocarcinoma-derived Caco-2 cells. The results showed that compounds 5 and 6 were adequately permeable in Caco-2 cells, with an estimated value of ≥ 5.15 log cm/s. Additionally, these compounds were anticipated to be substrates for P-glycoprotein, with predictive values between 0 and 0.3, suggesting bioavailability of ≥ 20% and 30%, respectively. These findings indicate that both compounds are expected to have favorable bioavailability profiles.

However, these two compounds exhibited substantial plasma protein-binding (PPB) characteristics, with predicted values below 90%. ADMET analysis revealed that these compounds showed efficient blood–brain barrier penetration, with empirical values of 0.9 to 1.0. Additionally, the unbound fraction in plasma for all compounds was greater than 5%, suggesting a favorable unbound state of the drugs in the bloodstream (Matta and Arabi 2011; Syahri et al. 2023; Vallianatou et al. 2022).

Several researchers, including Croce et al. (2022), Guo et al. (2008), Kwong and Wang (2020), Araújo et al. (2015), and Wang et al. (2022), have provided valuable insights into the mechanisms and toxicological effects of usnic acid on the liver, including mitochondrial dysfunction, oxidative stress, apoptosis, necrosis, genotoxicity, and teratogenicity. These findings highlights the need for a comprehensive evaluation of the safety profiles of usnic acid derivatives proposed as potential drug candidates for dengue virus inhibition. Structural modifications or alternative strategies should be carefully considered to minimize the risks of hepatotoxicity and carcinogenicity (Croce et al. 2022; Guo et al. 2008; Kwong and Wang 2020; Araújo et al. 2015; Wang et al. 2022).

**Figure 7:**
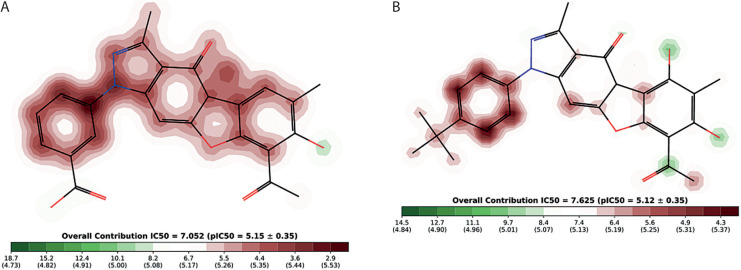
The human Ether-à-go-go-related gene (hERG) blocker for (**A**) compound 5 and (**B**) compound 6. Red contour regions indicate higher hERG blockage risk; green regions represent lower risk contributions

The ADMETLab 2.0 assessment for the human Ether-à-go-go-related gene (hERG) blocker yielded scores of 0.014 and 0.008 for compounds 5 and 6, respectively, which substantiates their safety profile. This finding was corroborated by the Pred-hERG analysis, which classified compounds 5 and 6 as non-cardiotoxic with a confidence level of 90% (Figure 7). The red dotted lines delineate the regions that adversely affect hERG blockade, where-as the contour lines with a vibrant green hue denote a greater positive influence of an atom on hERG blockade. Compounds 5 and 6 did not exhibit human hepatotoxicity (H-HT). Molecules classified within category 1 are deemed hepatotoxic (H-HT positive, +), whereas those in category 0 are non-hepatotoxic (H-HT negative, –). Similarly, the risk of drug-induced liver injury (DILI) was assessed as low, with a score of 0.986. Generally, drugs classified as category 1 pose a high risk of DILI, whereas those in category 0 exhibit no risk of DILI. Moreover, the Ames toxicity evaluation further confirmed the safety of compounds 5 and 6, as evidenced by their scores of 0.033 and 0.018, respectively. Drugs classified in category 1 are identified as Ames positive (+, toxic), while those in category 0 are considered Ames negative (–, non-toxic). Finally, the risk associated with oral toxicity was found to be low. The rat oral acute toxicity score of 0.11 classifies compounds 5 and 6 in category 0 (low toxicity), with category 1 compounds designated as highly toxic. Notably, as shown in Supplementary Table 1, all compounds exhibited reduced toxicity in terms of ADMET parameters and hERG blockers. These compounds were found to be substrates for both CYP1A2 and CYP2D6, suggesting their potential as promising candidates for anti-dengue therapy.

In the present study, ADMET predictions showed that all derivatives, particularly compounds 5 and 6, exhibited significantly improved safety profiles, although the parent compound, i.e., usnic acid, is known to induce hepatotoxicity through mechanisms such as oxidative stress, mitochondrial dysfunction, and apoptosis (Croce et al. 2022; Guo et al. 2008). These benefits could be attributed to the presence of pyrazole-based substitutions that affect metabolic pathways and reduce hepatotoxicity. Future studies should explore further structural changes to improve the pharmacokinetic properties of these derivatives, including increasing water solubility or decreasing lipophilicity to decrease hepatic retention. This approach may facilitate the development of safer usnic acid-based compounds for medical use.

## Conclusions

A computational investigation effectively predicted the efficacy of 10 usnic acid derivatives as potential inhibitors of dengue virus DENV-2 NS2B/NS3 serine protease. The findings revealed that compounds 5 and 6 exhibited binding free energies of –7.320 and –7.477 kcal/mol, respectively. Moreover, both these substances can form hydrogen bonds with the amino acid residue His51 in the active site of the protein 2FOM (derived from the PDB). DFT calculations also indicated that compounds 5 and 6 possess the most stable configurations, demonstrating the smallest energy gaps. The ADMET profile further validated their drug-like characteristics. These computational findings suggest that compounds 5 and 6 may serve as preliminary candidates for further investigation as inhibitors of dengue virus protease. However, experimental validation and comprehensive safety profiling are required before these compounds can be considered for drug development.

## Supplementary Material


