# Supplementary material for: Integrated analysis of usnic acid as a potential inhibitor of dengue NS2B/NS3 protease: DFT, molecular docking, MEP, ADMET, and drug-likeness evaluation
Source: BioTechnologia (Pozn). 2026 May 22;107(2):137–50. doi: 10.5114/bta/216303 (PMC13409369; doi:10.5114/bta/216303)
Supplement: Supplementary file 1 [file BTA-107-2-216303-s1.pdf]

**Supplementary Table 1.** Drug-likeness and absorption, distribution, metabolism, excretion, and toxicity (ADMET) analysis results

| Parameter | Panduratin A | 5 | 6 |
|---|---|---|---|
| Drug-likeness | | | |
| Molecular weight | 406.210 | 460.130 | 460.130 |
| H-bond acceptor | 4 | 9 | 9 |
| H-bond donor | 2 | 3 | 3 |
| LogP | 7.246 | 3.318 | 3.303 |
| TPSA | 66.760 | 138.950 | 138.950 |
| A (Absorption) | | | |
| Human intestinal absorption (HIA) | 0.011 | 0.073 | 0.090 |
| Caco-2 permeability (log cm/s) | –4.774 | –5.307 | –5.362 |
| P-glycoprotein inhibitor | 0.986 | 0.320 | 0.385 |
| P-glycoprotein substrate | 0.018 | 0.025 | 0.022 |
| $F_{20\%}$ | 0.883 | 0.031 | 0.043 |
| $F_{30\%}$ | 0.674 | 0.002 | 0.002 |
| D (Distribution) | | | |
| Plasma protein binding (PPB) (%) | 97.360 | 97.770 | 97.800 |
| Blood-brain barrier penetration (BBB) (cm/s) | 0.021 | 0.008 | 0.008 |
| Volume distribution (l/kg) | 1.584 | 0.594 | 0.655 |
| Fraction unbound (Fu) (%) | 4.645 | 1.635 | 1.634 |
| M (Metabolism) | | | |
| CYP1A2 substrate | 0.802 | 0.087 | 0.101 |
| CYP1A2 inhibitor | 0.817 | 0.041 | 0.041 |
| CYP2C19 substrate | 0.254 | 0.101 | 0.120 |
| CYP2C19 inhibitor | 0.968 | 0.044 | 0.048 |
| CYP2C9 substrate | 0.950 | 0.133 | 0.124 |
| CYP2C9 inhibitor | 0.945 | 0.495 | 0.635 |
| CYP2D6 substrate | 0.577 | 0.117 | 0.116 |
| CYP2D6 inhibitor | 0.931 | 0.020 | 0.039 |
| CYP3A4 substrate | 0.220 | 0.597 | 0.636 |
| CYP3A4 inhibitor | 0.840 | 0.099 | 0.127 |
| E (Excretion) | | | |
| Half time ($t_{1/2}$) | 0.061 | 0.711 | 0.779 |
| Clearance (ml/min/kg) | 12.251 | 0.630 | 0.666 |
| T (Toxicity) | | | |
| Human hepatotoxicity (H-HT) | 0.584 | 0.878 | 0.885 |
| hERG blockers | 0.272 | 0.014 | 0.008 |
| Rat oral acute toxicity | 0.092 | 0.822 | 0.768 |
| Ames toxicity | 0.041 | 0.033 | 0.018 |
| Drug induced liver injury (DILI) | 0.661 | 0.986 | 0.986 |
| Carcinogencity | 0.137 | 0.763 | 0.742 |

*hERG* – human ether-á-go-go-related gene, TPSA – topological polar surface area.
